# Working capacity level defines the specific impairment profile of the comprehensive ICF core set for multiple sclerosis

**DOI:** 10.1038/s41598-025-87827-6

**Published:** 2025-01-28

**Authors:** Daiva Valadkevičienė, Irena Žukauskaitė, Indre Bileviciute-Ljungar, Rasa Kizlaitienė, Dalius Jatužis, Virginija Danylaitė Karrenbauer

**Affiliations:** 1 Clinic of Neurology and Neurosurgery, Institute of Clinical Medicine, Faculty of Medicine, Vilnius University, Vilnius, Lithuania; 2 The Agency for the Protection of the Rights of Persons with Disabilities under the Ministry of Social Security and Labor of the Republic of Lithuania (APRPD), Previously the Disability and Working Capacity Assessment Office, under the Ministry of Social Security and Labour of the Republic of Lithuania (DWCAO), Vilnius, Lithuania; 3 Institute of Psychology, Faculty of Philosophy, Vilnius University, Vilnius, Lithuania; 4 Department of Clinical Science, Karolinska Institutet, Stockholm, Sweden; 5 Multidisciplinary Pain Clinic, Capio St. Göran Hospital, Stockholm, Sweden; 6 Department of Clinical Neuroscience, Karolinska Institutet, Stockholm, Sweden; 7 Medical Unit Neuro, Karolinska University Hospital, Stockholm, Sweden

**Keywords:** Multiple sclerosis (MS), International classification of functioning, disability and health (ICF), Comprehensive ICF core set for multiple sclerosis, Working capacity level, Disability, Neurology, Multiple sclerosis, Outcomes research

## Abstract

Multiple sclerosis (MS) unfavorably affects working capacity. The Comprehensive International Classification of Functioning, Disability and Health Core Set for MS (cICF-MS), issued by the World Health Organization, has not yet been extended to evaluate working capacity level (WCL). To evaluate the relative importance of cICF-MS categories in relation to WCL. Persons with MS (PwMS), *N* = 129, who had been referred to Lithuania’s Disability and Working Capacity Assessment Office for WCL determination, were divided into three groups according to the percentage of remaining WCL (WCL1 had 0–25%, WCL2 had 30–40%, and WCL3 had 45–55%). Data regarding the cICF-MS categories were collected through telephone interviews and patient documentation. Using the fractional ranking method, the mean values of cICF-MS impairment were ranked from the most severely affected to the least affected (rank 1–93). Ranks with the 10 highest mean values of impairment severity in each WCL group were included in a descriptive analysis. In the WCL1 and WCL2 groups, the most-affected cICF-MS categories reflected disability related to gait and motor function. The WCL3 group presented with pain, fatigue, and impairments to visual acuity, psychic stability, urination, and memory. This study has identified specific cICF-MS impairment profiles based on remaining WCL.

## Introduction

Multiple sclerosis (MS) is an inflammatory and degenerative disease of the central nervous system (CNS) that affects the work capacity of young people of working age^1^. MS causes various symptoms, including motor function impairment, fatigue, pain, sensory and visual disturbances, and cognitive deficits^1^, and is associated with reduced working capacity and lower earnings^2^. The most common cognitive deficit is reduced processing speed, which is also associated with lower income^3^ and has been found to predict the deterioration of employment status 1 and 3 years in the future^4^.

As an individual’s Expanded Disability Status Scale (EDSS) score increases, their workforce participation decreases, and their workplace productivity is increasingly affected by fatigue and impaired cognition^5^. A European cross-sectional study that included more than 16,000 individuals from 16 European countries from 2015 to 2016 demonstrated that workforce participation had decreased rapidly with advancing EDSS scores: 82% of healthy individuals with an EDSS score of 0, and only 8% among PwMS, with an EDSS score of 9, were able to work^6^.

In 2005, 61.7% of PwMS in Sweden were receiving a partial or full disability pension, compared to 14.2% of controls^7^. Furthermore, a study in the USA reported that 21.4% of employed PwMS took at least one sick leave absence during a 1-year period^8^. Moreover, the incidence of MS in Lithuania was 6.5 per 100,000 from 2001 to 2015, and it was predicted to increase to 13 per 100,000 by 2020^9^.

The International Classification of Functioning, Disability, and Health (ICF) is the framework used by the World Health Organization (WHO) for measuring health and disability at both the individual and population levels. At the 54th World Health Assembly on May 22, 2001, the ICF was officially endorsed by all 191 WHO member states as the international standard for describing and measuring health and disability in a given environmental context (Resolution WHA 54.21)^10^. It has been designed to be used as a mutual language for communication among the groups that work to ensure ideal outcomes for PwMS, namely health professionals, researchers, patients, patient organizations, and policymakers. The main categories of the Comprehensive and Brief ICF core sets were established at the 2011 International Consensus Conference^11^.

The Comprehensive ICF-MS (cICF-MS) and Brief ICF-MS are seldom-used tools for evaluating PwMS’ disability and problem profiles. Only a limited number of studies (*n* = 20) regarding the topics of “ICF core set” and “multiple sclerosis” could be found in the PubMed database (https://pubmed.ncbi.nlm.nih.gov/) as of a search date of November 1, 2023.

The ICF is useful for a broad spectrum of applications, such as social security, evaluation in managed health care, and population surveys at the local, national, and international levels. It offers a conceptual framework for information that applies to personal health care, including prevention, health promotion, and the improvement of participation through removing or mitigating societal hindrances and encouraging the provision of social support and facilitators. It is also useful for studying healthcare systems in terms of both evaluation and policy formulation^10^.

In this observational, prospective cohort study, we aimed to map the cICF-MS into “Body functions” and “Activities and participation” categories and evaluate the relative importance of the cICF-MS categories in relation to a reduced work capacity level (WCL) in PwMS. We proposed the following hypothesis:

Hypothesis: Different WCL groups have specific profiles for the most affected cICF-MS categories.

## Results

### Study cohort

In total, 129 PwMS fulfilled the inclusion criteria. The clinicodemographic characteristics are presented in Table 1.

**Table 1:** Clinicodemographic characteristics of the study cohort.

Variables		Whole cohort *N* = 129	WCL1 n = 33	WCL2 n = 85	WCL3 n = 11	p
Age, mean (SD)^a^		49.1(10.5)	50.6 (9.5)	49.3 (10.5)	42.9 (11.5)	.100
Age at onset, mean (SD)^a^		35.3 (12.2)	33.1 (11.6)	36.7 (12.6)	31.3 (8.8)	.192
Age at diagnosis, mean (SD)^a^		37.7 (11.7)	36.9 (10.7)	38.7(12.2)	32.2 (9.3)	.205
Baseline EDSS, mean (SD)^a^		4,6 (1,3)	6,0 (1,1)	4,2 (0,8)	2,8 (0,5)	< .001
Gender^b^	Male	41 (32%)	7 (21%)	32 (38%)	2 (18%)	.136
Education^b^	Basic	7 (5%)	2 (6.1%)	5 (6%)	0 (0.0%)	.739
	Secondary	15 (12%)	4 (12%)	8 (9%)	3 (27%)	
	Vocational	33 (26%)	9 (27%)	21 (25%)	3 (27%)	
	College	25 (19%)	8 (24%)	16 (19%)	1 (9%)	
	Higher	49 (38%)	10 (30%)	35 (41%)	4 (36%)	
Profession^b^	(has)	107 (83%)	27 (82%)	72 (85%)	8 (73%)	.598
Employment^b^	(yes)	62 (48%)	7 (21%)	47 (55%)	8 (73%)	.001
Disease type^b^						
	SPMS	15 (12%)	10 (30%)	5 (6%)	0 (0%)	.001
	PPMS	7 (5%)	2 (6%)	5 (6%)	0 (0%)	
	RRMS	107 (83%)	11 (64%)	75 (88%)	11 (100%)	
DMTs^b^						
Moderate efficacy		59 (45.7%)	10 (30.3%)	42 (49.4%)	7 (63.6%)	< .001
High efficacy		48 (37.2%)	9 (27.3%)	36 (42.4%)	3 (27.3%)	
Untreated		22 (17%)	14 (42%)	7 (8%)	1 (9%)	

*DMTs* disease-modifying treatments, *NA* information unavailable, *N* number of individuals, *PPMS* primary-progressive multiple sclerosis, *RRMS* relapsing–remitting multiple sclerosis, *SPMS* secondary-progressive multiple sclerosis, *WCL1* working capacity level group 1 (with the lowest working capacity level), *WCL2* working capacity level group 2 (with an intermediate work capacity level), *WCL3* working capacity level group 3 (with the highest working capacity level).

^a^compared using the Mann–Whitney test.

^b^compared using the chi-square test.

### Ranking of ICF components in the WCL groups

**Fig. 1:**
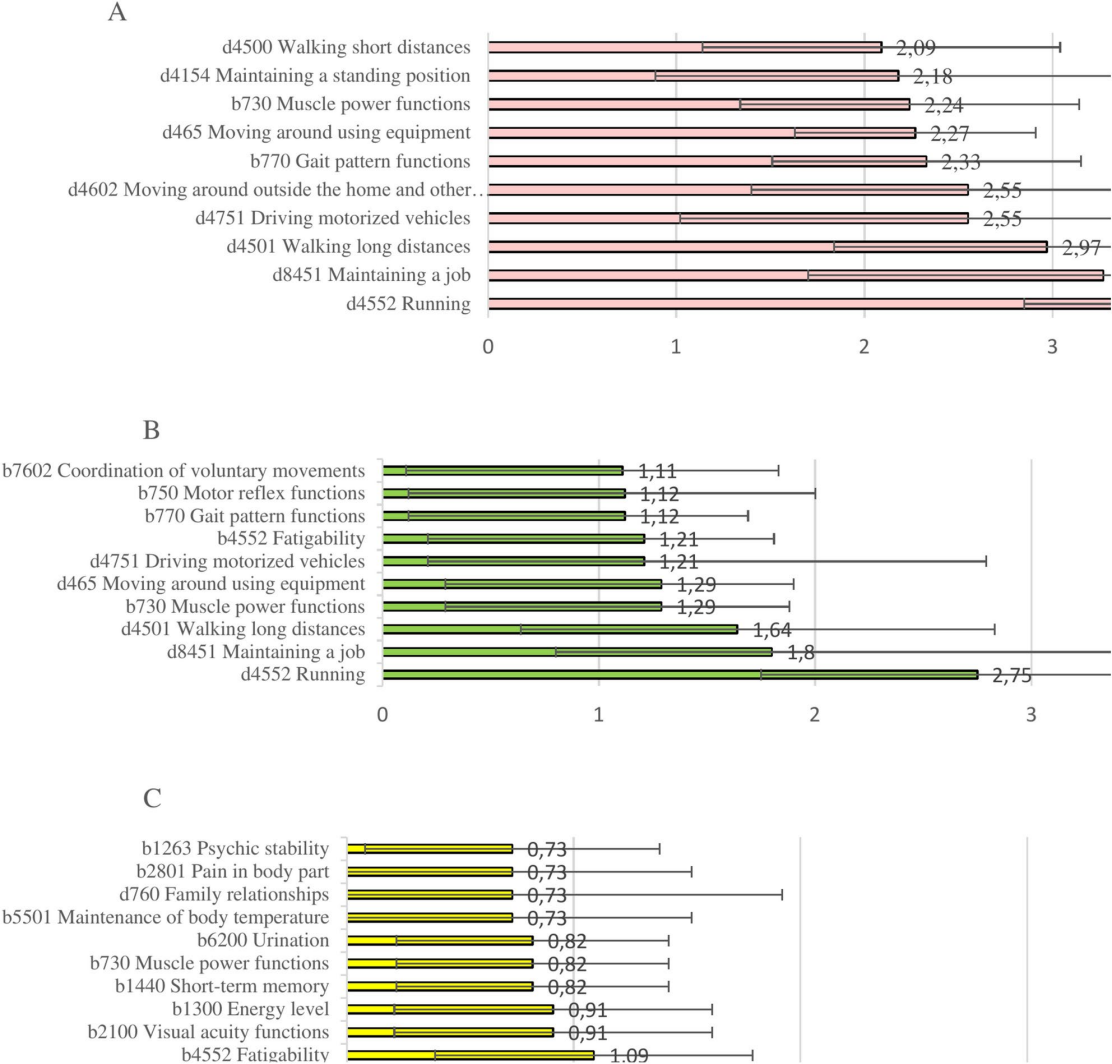
Top 10 most-affected categories according to cICF-MS score, ranging from 0 to 4 in the WCL1 (**A**), WCL2 (**B**), and WCL3 (**C**) cohorts. The graph plots the mean and standard deviation of the top 10 qualifiers sorted from least-affected/impaired (rank 10) to the most-affected/impaired (rank 1). ICF qualifiers were evaluated according to the following scale: 0 = no impairment/difficulty; 1 = mild impairment/difficulty; 2 = moderate impairment/difficulty; 3 = severe impairment/difficulty; and 4 = complete impairment/difficulty.

The ranking of the top 10 ICF categories in the WCL groups is presented in Supplementary Table 1. An overview of the top 10 categories in each WCL group is presented in Fig. 1A,B,C. The distribution and severity of ICF categories in the “Body functions” and “Activities and participation” sections are presented in Fig. 1.

*The top 10 ranked ICF mean value list contains 13 ICF variables because several ICF categories shared the same ICF mean values and had the same rank.

### Categories, specific for each WCL group

For the WCL1 and WCL2 groups, many cICF-MS categories were related to walking and motor functions, while WCL3 presented with pain, fatigue, and impairments to visual acuity, psychic stability, urination, memory. The WCL group specific ICF category lists are presented in Fig. 1 and Supplementary Table 2.

### Affected categories, general for all three WCL groups

**Fig. 2:**
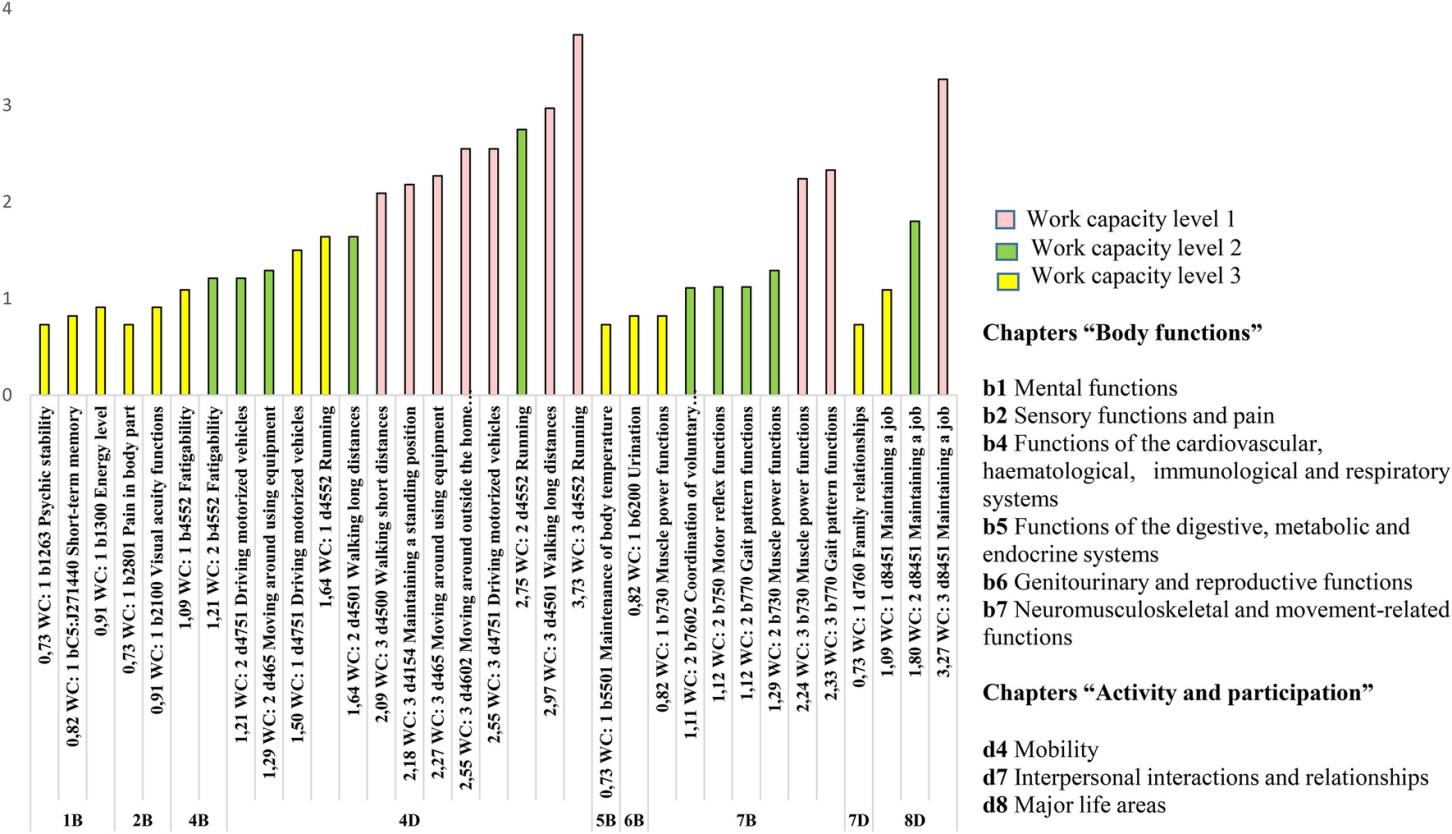
Distribution and severity of ICF categories mean value in the “Body functions” and “Activities and participation” chapters.

The following cICF-MS categories were affected in all three WCL groups: d4552 Running (the highest ranked as most severely affected category in all three WCL groups), d4751 Driving motorized vehicle, d8451 Maintaining a job, and b730 Muscle power functions (Figure 2 and Supplementary Table 2).

## Discussion

This is the first explorative study to have assessed the most-affected cICF-MS categories in a consecutively recruited cohort of PwMS with a reduced WCL, referred for evaluation of current working capacity. The study has identified differences in cICF-MS impairment between groups defined by WCL. The WCL1 and WCL2 groups had the highest number of affected cICF-MS categories related to different aspects of gait and motor dysfunction, whereas the WCL3 group presented cICF-MS with pain, fatigue, and impairment related to dysfunction of different functional systems, such as visual acuity, psychic stability, urination, and memory. The most severely affected ICF categories common for all three WCL groups were the following: d4552 Running, d8451 Maintaining a job, d4751 Driving motorized vehicles, and b730 Muscle power functions.

### Acknowledgement of biases

We acknowledge that the single-center and unblinded nature of the study presents the potential for bias. However, we evaluated these aspects as having a low impact due to the relatively high number of participants and the unavailability of an entire clinicodemographic dataset during the telephone interviews.

### Telephone interview versus physical visit

Due to legal restrictions during the COVID-19 pandemic^12^, the data were collected through telephone interviews rather than in-person interviews. According to the literature, the quality and richness of the data obtained through telephone interviews are comparable to those of data obtained through personal interviews^13^. We believe that our data collection method did not affect the study results.

### Selection bias

We assessed only patients who had ongoing working capacity assessments at the DWCAO (those with a reduced WCL). No patients in the current study exhibited a WCL of 60–100%, nor were there any healthy controls. Populations were preselected based on the study setting and because the patients who had been referred to the DWCAO for WCL determination already had a disability. The PwMS were recruited consecutively as they visited the DWCAO, provided consent for the study, and fulfilled the inclusion criteria. The study thus reflects the most-impaired ICF categories only in PwMS with a reduced working capacity.

### Sex representation in the study

We acknowledge that the present study included twice as many females (68.2%) as males (31.8%). According to epidemiological data, this distribution reflects the sex ratio prevalent in current MS cases in Lithuania (MS-in-EU-access.pdf [emsp.org]). This MS prevalence difference is thought to stem from immune response differences and the differences in the pathophysiology of the CNS damage due to sexual dimorphism that affects disease susceptibility and progression^14^.

### Limitations

The ICF components of “Environment” and “Body structure” were not evaluated due to study protocol constraints, representing a limitation of this study. Nevertheless, the study setting, and origin involved a targeted task of searching for relatively important cICF-MS categories in the “Activities and participation” and “Body functions” components, in patients groups, stratified according to the WCL.

### Interpretation of the results

The highest number (*n* = 13 of 33 ranked ICF categories) of the most severely affected categories of the cICF-MS were from Chapter d4 (Mobility; Fig. 2). Differential representation in category distribution between WCL groups was observed: while the WCL3 group had only two impaired categories, d4552 Running and d4751 Driving motorized vehicles, WCL1 and WCL2 shared impaired cICF-MS categories that reflected different aspects of PwMS’ capacity to move, including d4501 Walking long distances, d4500 Walking short distances, and d465 Moving around using equipment. Categories that were impaired explicitly in WCL1 in this chapter were d4602 Moving around outside the home and d4154 Maintaining a standing position. This specific distribution of cICF-MS in WCL groups could be attributed to different disability levels: WCL1 exhibited a mean EDSS of 6.0 (reduced walking ability and need of unilateral walking aid), WCL2 had a mean EDSS of 4.4 (might have restricted walking distance), and WCL3 had a mean EDSS of 2.8 (no restriction of waking distance; Table 1). We underscore the importance of reviewing PwMS’ ability to move and, if possible, finding personalized solutions and compensatory strategies by consulting physiotherapists and occupational therapists for PwMS with reduced WCLs who intend to continue to work or aim to increase their workload.

From the same chapter, 4d (Mobility), cICF-MS category d4552 Running achieved rank 1 and was the most severely affected ICF category in all three WCLs. Running is a complex activity involving the coordinated actions of many parts of the CNS and many muscles^15^. In PwMS, diseases causes degeneration of the neuromuscular system resulting in motor control deficits, reduced movement efficiency, and can manifest as gait and running impairments^16^.

The category d4751 Driving motorized vehicles from chapter 4d (Mobility) was impaired across all three WCL groups. In Lithuania, it is possible to compensate for patients’ difficulties in using transportation and to potentiate patient mobility in society by providing personal assistance services for up to 4 h per day, 7 days per week. This legal right of patients supports their ability to use transportation, their mobility, their social relationships, and their personal hygiene^17^. In addition, in Lithuania, there is cost reimbursement for purchasing and adapting a car as well as discounted tickets for buses, trolleybuses, trains, and ferries^18^. In Sweden, individuals can use either patient transportation services (*Färdtjänst*)^19^ or taxi trips to and from work as part of sick leave conditions^20^ for cases in which only a single obstacle—a trip by bus or one’s own car—causes the patient to remain at home and not attend their job. While the number of patients who have received economic compensation for taxi trips to and from their job in Sweden is low, it is gradually increasing. For example, 2737 patients in 2007 and 3384 patients in 2013 received compensation for work-related taxi trips^20^. According to the Swedish Social Insurance Inspectorate (Inspektionen för socialförsäkringen; ISF), both the number and length of sick leave absences could be reduced if more patients would use this benefit^20^.

The WCL3 group, possessing the highest working capacity, included the PwMS with the lowest mean EDSS (2.8). Compared to groups WCL1 and WCL2, WCL3 reported higher impairment of the following cICF-MS items: b4552 Fatigability, b1300 Maintaining energy levels, b2100 Visual acuity functions, b1400 Short-term memory, b6200 Urination, b1263 Psychic stability, b2801 Pain in body part, b5501 Maintenance of body temperature, and d760 Family relationships. Some of these cICF-MS items, namely visual function, urinary bladder and bowel function, and higher mental functions (including assessment of fatigue, memory, and depression), resemble EDSS functional systems that are evaluated and contribute to the final EDSS score for patients without walking distance restrictions^21^. The broad spectrum of affected cICF-MS items in the “Body function” chapter pinpoints the need for support from different specialist physicians (e.g., ophthalmologists, urologists, neurologists, psychiatrists), psychologists and social workers, and occupational therapists to address impaired functions to maintain current working capacity.

As illustrated in Fig. 2, the most impaired category present in all three WCL groups was d8451 Maintaining a job. This category had the highest mean severity values in WCL1 (the group with the lowest working capacity). Patients on sick leave had difficulties maintaining their jobs in the long term. In a Swedish case–control study that included 6,092 PwMS, the mean annual prevalence of sick leave ranged from 12 to 23%, while that of disability pension (i.e., permanent loss of working capacity) ranged from 12 to 55%, depending on the time since diagnosis^22^.

A limited number of statistical analyses to evaluate the clinicodemographic differences between the WCL groups did not influence the study outcomes.

In a recent publication from the Netherlands, de Wind et al. have identified the ICF Core Set for the assessment of working capacity and guidance in return for work using the Delphi consensus method among occupational health professionals^23^. These authors created a list of 20 ICF items from the category “Activities and participation” reflecting the minimal criteria necessary to evaluate a patient’s potential to return to work. We compared the set of ICF items from the “Activities and participation” chapter between the current study and that of de Wind et al.^23^ and found that only four of their ICF core set items were present in our study, namely d475 Driving, d450 Walking, d415 Maintaining a body position, and d410 Changing basic body position. At the Delphi consensus conference held by de Wind et al.^23^, 16 other ICF items were agreed upon by experts as important for patients on sick leave; however, these ICF items were absent from the current study (e.g., d120 Other purposeful sensing, d177 Making decisions, d110 Watching, d159 Basic learning, 240 Handling stress and other psychological demands, d115 Listening, d451 Going up and down stairs, d740 Formal relationships, d160 Focusing attention, and d430 Lifting and carrying objects). Some possible explanations for the differences between the studies are as follows:

- (1) A mismatch may exist among expert opinions regarding which ICF items influence the WCL and individual patient assessments using ICF items (patient-reported and treating-physician-reported data were used in the current study).
- (2) Possible differences may exist in biopsychosocial perspectives in studies originating from different countries.
- (3) The current study examined patient-reported, MS-specific ICF items, whereas the other study reported expert consensus opinions regarding ICF item collection without disease specification.

Currently known the single case of usage of ICF application in sick leave procedural administration is in Sweden: The Swedish Social Insurance Agency already uses ICF items on sick leave forms to describe PwMS’ impairment of body functions and activity limitations^24^. The information source and the selection algorithm used for ICF were not available on the open web pages of the National Board of Health and Welfare (https://roi.socialstyrelsen.se/fmb/multipel-skleros/128); however, current sick leave forms contain lists of 23 suggested multiple-choice ICF items to describe patients’ “Activities and participation” (e.g., reading, decision making, social interaction, and writing) as well as 23 items to describe “Body functions” (e.g., energy levels, higher cognitive functions, attention, and vision). Sick leave forms are issued by the treating physician or neurologist and certify that the patient has reduced work capacity by 25, 50, 75 or 100%. If an individual’s workability status is not improving during the 1–3-year period, the Swedish Social Insurance Agency may suggest an investigation to determine permanent disability of 25, 50, 75, or 100%. For the final decision regarding permanent disability, which is made by Swedish Social Insurance Agency, a certificate issued by the treating physician is required. In conflicting cases, doctors employed by the Swedish Social Insurance Agency are consulted.

### Clinical and demographic factors correlated with job-related difficulties

A study by Cataneo et al. that included 105 PwMS and 20 healthy subjects showed that participation restrictions were present in PwMS and increased with disability level. In the study, 77% of participants showed participation restrictions. In group of PwMS with EDSS < 4, 40% had participation restrictions; In group PwMS with EDSS > 5.5, 82% had participation restrictions. Cognitive disorders were more associated with participation restrictions than physical limitations^25^.

A European study showed that 31–65% of PwMS were employed^6^. Disease duration, degree of disability, level of education, and type of work were factors influenced the employability of PwMS^26,27^.

In 2020, the proportion of PwMS who were employed varied significantly, ranging from 20% in Belarus to 50% in Italy and Ireland, 65% in Switzerland and Turkey, and 76% in Slovenia, according to the MS Barometer^28^.

In a study by Ellenberger et al., using data from the European Registry in Multiple Sclerosis (EUReMS) for four European Countries, the authors found that disease-related variables such as disability progression, low quality of life, and being close to the statutory retirement age were the factors that contributed most to permanent working capacity loss. Apart from demographic and disease-related issues, country-specific factors may also impact employment status^29^.

In another study by Börsch-Supan et al., the authors have been studying the interrelated roles of health and welfare state policies in the decision to take up disability insurance (DI) benefits due to work disability (WD), defined as the inability to engage in employment as a result of illness^30^. The study included 12 European countries and the USA. This study showed that the mismatch between WD and received DI benefits varies greatly across countries. Health variables explained a substantial share of the within-country variation in DI. Most of the variation between countries was explained by differences in DI policies^30^.

### Generalizability and external validity of the results

The results can only be generalized to members of the Lithuanian MS population who have disabilities and reduced working capacity and visit the DWCAO. Due to the limited sample size and because the population was preselected according to the inclusion criteria, the generalizability of the study results is low^31^; moreover, we are unable to generalize our findings to the entire MS population. The external validity of the study is poor^32^ because the cohort was restricted to only a specific subgroup of PwMS (i.e., those with reduced working capacity).

The results of this study lay the foundation for the broader application of ICF-MS at individual and group levels both to identify and shape individual patient compensatory strategies and to make amendments at the societal level to maintain work capacity in PwMS. This study must be repeated using larger cohorts and in international, multicenter study settings to determine whether the patterns of ICF impairment are reproducible.

## Materials and methods

### Study design and setting

This cross-sectional study was conducted at the Clinic of Neurology and Neurosurgery of the Institute of Clinical Medicine (Faculty of Medicine, Vilnius University) and at the Disability and Working Capacity Assessment Office (DWCAO) under the Ministry of Social Security and Labor of the Republic of Lithuania.

The study was part of a postdoctoral fellowship titled “Value of International Classification of Functioning, Disability and Health for evaluating disability for people with multiple sclerosis in Lithuania” under Measure No. 09.3.3-LMT-K-712-23-0180, which was authored by the fellow Dr. Daiva Valadkevičienė. The study was conducted after contract No. (5.74) SU-2990 was signed between Vilnius University and the DWCAO on November 17, 2021.

This was a prospectively recruited cohort follow-up study. Patients of both sexes were asked to participate after receiving a WCL assessment at the DWCAO. Patients who provided written informed consent and fulfilled the inclusion criteria were recruited. The neurologist assessed PwMS by telephone interview using the cICF-MS. Legal limitations for face-to-face interviews existed during the data collection period due to COVID-19 pandemic restrictions imposed by the Lithuanian Government on November 4, 2020^12^. Data collection was terminated on August 1, 2022.

The report was written according to STROBE guidelines^33^.

### Participants

In total, 129 consecutive participants with MS participated in the study. The inclusion criteria were as follows:

1. Aged older than 18 years but not older than retirement age, with an evaluated disability for MS at DWACO;
2. MS diagnosis according to the McDonald criteria 2017 revisions^34^;
3. MS in remission and a stable neurological condition;
4. Fluent in the Lithuanian language;
5. Provided voluntary consent to participate in the study, as certified by the participant’s signature on an informed consent form.

The exclusion criteria were as follows:

1. Patients who did not fulfill the inclusion criteria;
2. Patients who were not compliant with the study protocol.

**Fig. 3:**
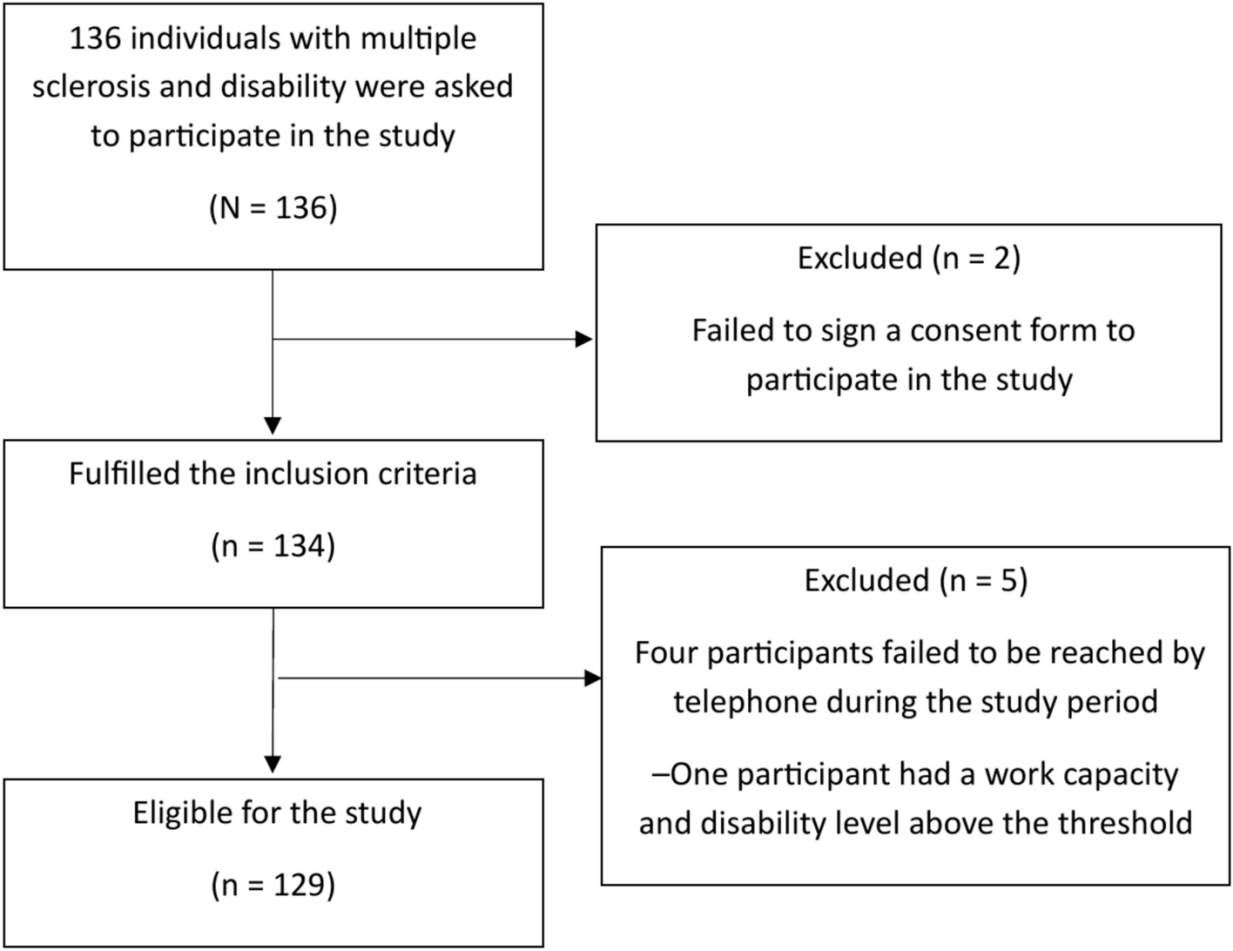
Flowchart of the study cohort selection process.

Figure 3 presents a flowchart of the included individuals and the reasons for exclusion:

### Definitions

#### Immunomodulatory treatment efficacy

According to the revised guidelines of the Association of British Neurologists for prescribing disease-modifying treatments in MS, disease-modifying treatments were divided into high-efficacy (alemtuzumab and natalizumab) and moderate-efficacy (dimethyl fumarate, fingolimod, glatiramer acetate, IFN-β preparations, and teriflunomide) treatments^35^. Cladribine, ofatumumab, rituximab, ocrelizumab, and ozanimod were clustered as high-efficacy treatments according to a network meta-analysis of the annualized relapse rate^36^.

#### Secondary diagnoses

Secondary diagnoses were coded according to the International Statistical Classification of Diseases and Related Health Problems, 10th revision, Australian Modification (ICD-10-AM)^37^. Table 2 presents the number of patients with secondary diagnoses as well as the ICD codes.

**Table 2:** ICD-10-AM codes and the number of patients with secondary diagnoses.

ICD-10-AM Code	Diagnosis in Text	Number of patients with a diagnosis in the study cohort
I11	Hypertensive heart disease	1
E03.2	Hypothyroidism due to medicaments and other exogenous substances	1
H47.2	Other disorders of the optic second nerve and visual pathways	2
E03.8	Other specified hypothyroidism	1
F20	Paranoid schizophrenia	1
F32.1	Major depressive disorder, single episode, moderate	1
F33.1	Major depressive disorder, recurrent, moderate	1
H17.8	Corneal scars and opacities	1
H20	Iridocyclitis	1
I11.9	Hypertensive heart disease without heart failure	5
I50	Heart failure	1
R32	Unspecified urinary incontinence	1

*ICD-10-AM* International statistical classification of diseases and related health problems, 10th revision, Australian modification.

## Methods

### Data collection

The primary investigator collected data on “Body functions” and “Activities and participation” through telephone interviews as well as from patient documentation. The documentation was provided to the DWACO by the treating physician, who had referred the patients to the DWACO to determine the WCL.

### Definition of impairment magnitude in ICF categories

The extent or magnitude of ICF category impairment was determined according to the instructions in the online ICF book^10^. The instructions are as follows:

0 = NO impairment (none, absent, negligible): a reduction of function by 0–4%;

1 = MILD impairment: slight or low reduction of function by 5–24%;

2 = MODERATE impairment: medium or fair reduction of function by 25–49%;

3 = SEVERE impairment: high or extreme reduction of function by 50–95%;

4 = COMPLETE impairment: total reduction in function by 96–100%

### WCL determination for PwMS at the DWCAO

The WCL was established according to by describing the criteria and procedures for determining the WCL approved by the order of the Ministers of Social Security, Labor, and Health after a complex assessment of the medical criteria (i.e., the person’s basic work capacity) and the criteria of the person’s activities and ability to participate. WCL refers to the “ability of a person to implement a professional competency previously gained, to acquire a new professional competency, or to perform jobs that require lower professional competency^38^”. The WCL is established for people from 18 years of age to the age of retirement by assessing not only medical but also functional, professional, and other criteria that impact work capacity.

The WCL is set at intervals of 5% by legal regulations regarding the process of setting the WCL. Such regulations are decided by the Disability and Capacity for Work Service under the Ministry of Social Security and Labor^39^.

“In Lithuania, all persons who have been assessed at 0–55% of working capacity are designated “persons with disabilities” and are guaranteed legislatively determined benefits according to this status. The lower the percentage of working capacity scores, the more severe the disability. Work capacity is evaluated in five-percentage-point intervals, ranging from 0 to 100 (where 0–25% indicates total incapacity for work, 30–55% indicates a partial capacity for work, and 60–100% indicates that a person is capable of working). The assessment consists of (i) medical criteria (hereafter basic work capacity) that are adjusted by a coefficient created from (ii) a person’s activity and ability to participate as assessed by the Questionnaire of the Individual’s Activity and Ability to Participate^18^”. The detailed WCL assessment in Lithuania procedure protocol is described in World Bank and European Commission report^18^.

A person of working age is determined to have a disability when their WCL is 55% or less. In the present study, PwMS with working capacities ranging from 0 to 25% were clustered into WCL group 1 (WCL1), patients with intermediate working capacities ranging from 30 to 40% were clustered into WCL group 2 (WCL2), and patients with the highest working capacities ranging from 45 to 55% were clustered into WCL group 3 (WCL3).

### Fractional ranking of ICF components in the WCL groups

To evaluate which ICF components were negatively affecting the well-being of the patients the most, all scores for each ICF category (0: no impairment or difficulty; 1: mild; 2: moderate; 3: severe; and 4: complete impairment or difficulty) in each WCL group were summed and divided by the number of group members. All categories were then sorted from the highest average score (rank 1) to the lowest average score (rank 93; data not shown) using the fractional ranking or mean method^40^; fractional ranking was used to rank items that had equal values. Items with equal values were assigned the average of their positions in the ascending (or descending) order of the figures^40^.

Only the top 10 ranked categories for each WCL group were included in the analysis, in which rank 1 included the most-affected cICF category in the WCL group and rank 10 included the least-affected cICF categories between ranks 1 and 10.

In the current study, four cICF-MS categories in the WCL3 group received the same mean score of 0.73 in descending order of cICF mean values in positions 10, 11, 12, and 13 (Supplementary Table 1). Using the fractional ranking method, all four of these categories received the mean rank of 10.5, and all four shared the 10th rank place. For this reason, the WCL3 group included 13 cICF categories, ranked between 1 and 10.

### Graphical presentation of ICF categories, clustered in chapters

The ICF categories were grouped according to ICF chapter origin as follows: “Body functions” (b1, b2, b4, b5, b6, and b7) and “Activities and participation” (d4, d7, and d8). The ICF one-level classification is presented in the ICF online book. The categories are presented graphically in Fig. 2.

### Statistical analysis

Variables were compared between the groups using Mann**–**Whitney and chi-square tests. Because the patients had been recruited consecutively, all of the clinicodemographic data were available, and we collected the cICF-MS data consecutively. Thus, a complete data set containing no missing data was obtained.

### Ethical considerations

This study was approved by the Lithuanian Bioethics Committee (No. 2021/10-1387-855) on October 26, 2021. Each study participant signed an informed consent form before participating. The study was conducted following the principles of the Declaration of Helsinki^10^.

### Sex and gender equity in research (SAGER)

This study complied with the SAGER guidelines during data collection, analysis, and report writing^41^.

## Supplementary Information

Supplementary Information.

## Data Availability

The data that support the findings of this study are not openly available due to reasons of sensitivity and are available from the primary investigator and co-author Daiva Valadkevičienė upon reasonable request. Data are located in controlled access data storage at Vilnius University.
