# Supplementary material for: Working capacity level defines the specific impairment profile of the comprehensive ICF core set for multiple sclerosis
Source: Sci Rep. 2025 Jan 28;15:3547. doi: 10.1038/s41598-025-87827-6 (PMC11775219; doi:10.1038/s41598-025-87827-6)
Supplement: Supplementary file 1 — Supplementary Information. [file 41598_2025_87827_MOESM1_ESM.pdf]

Supplementary materials

WORKING CAPACITY LEVEL DEFINES THE SPECIFIC IMPAIRMENT PROFILE OF THE COMPREHENSIVE ICF CORE SET FOR MULTIPLE SCLEROSIS

Daiva Valadkevičienė[1,2§], Irena Žukauskaitė[3§], Indre Bileviciute-Ljungar[4,5], Rasa Kizlaitienė[1], Dalius Jatužis[1], Virginija Danylaitė Karrenbauer [6,7*]

Supplementary Table 1. Top 10 Rankings of the most severely affected categories of cICF-MS in the WCL group (rows in pink signifies the group with the least work capacity level, WCL1; rows in green signifies the intermediate work capacity level, WCL2; rows in yellow signifies the highest work capacity level, WCL3).

ICF Category	Mean	SD	Rank in WCL group	Chapter	WCL
**d4552 Running**	3,73	0,88	1,00	d4	1
**d4552 Running**	2,75	1,45	1,00	d4	2
**d4552 Running**	1,64	1,29	1,00	d4	3
**d4751 Driving motorized vehicles**	1,5	2,07	2,00	d4	3
**d8451 Maintaining a job**	3,27	1,57	2,00	d8	1
**d8451 Maintaining a job**	1,8	1,99	2,00	d8	2
**d4501 Walking long distances**	2,97	1,13	3,00	d4	1
**d4501 Walking long distances**	1,64	1,19	3,00	d4	2
**b4552 Fatigability**	1,09	0,7	3,50	b4	3
**d8451 Maintaining a job**	1,09	1,87	3,50	d8	3
**d4751 Driving motorized vehicles**	2,55	1,53	4,50	d4	1
**d4602 Moving around outside the home and other buildings**	2,55	1,15	4,50	d4	1
**d465 Moving around using equipment**	1,29	0,61	4,50	d4	2
**b730 Muscle power functions**	1,29	0,59	4,50	b7	2

**b1300 Energy level**	0,91	0,7	5,50	b1	3
**b2100 Visual acuity functions**	0,91	0,7	5,50	b2	3
**b770 Gait pattern functions**	2,33	0,82	6,00	b7	1
**b4552 Fatigability**	1,21	0,6	6,50	b4	2
**d4751 Driving motorized vehicles**	1,21	1,58	6,50	d4	2
**d465 Moving around using equipment**	2,27	0,64	7,00	d4	1
**b1440 Short-term memory**	0,82	0,6	8,00	b1	3
**b6200 Urination**	0,82	0,6	8,00	b6	3
**b730 Muscle power functions**	2,24	0,9	8,00	b7	1
**b730 Muscle power functions**	0,82	0,6	8,00	b7	3
**b770 Gait pattern functions**	1,12	0,57	8,50	b7	2
**b750 Motor reflex functions**	1,12	0,88	8,50	b7	2
**d4154 Maintaining a standing position**	2,18	1,29	9,00	d4	1
**d4500 Walking short distances**	2,09	0,95	10,00	d4	1
**b7602 Coordination of voluntary movements**	1,11	0,72	10,00	b7	2
**b1263 Psychic stability**	0,73	0,65	10,50	b1	3
**b2801 Pain in body part**	0,73	0,79	10,50	b2	3
**b5501 Maintenance of body temperature**	0,73	0,79	10,50	b5	3
**d760 Family relationships**	0,73	1,19	10,50	d7	3

Abbreviations: SD, standard deviation; WCL 1, work capacity level 1-MS patients with the lowest work capacity; WCL 2, work capacity level 2, MS patients with intermediate working capacity. WCL3, work capacity level 3- MS patients with the highest work capacity.

Supplementary Table 2. Distribution of top 10 ranked cICF-MS categories: common for all three WCL groups and specific for each WCL group

WCL group	cICF-MS categories common for all WCL groups	cICF-MS categories specific for each WCL group
WCL1	d4552 Running<br>d4751 Driving motorized vehicles<br>d8451 Maintaining a job<br>b730 Muscle power functions	d4501 Walking long distances<br>d4602 Moving around outside the home and other buildings<br>b770 Gait pattern functions<br>d465 Moving around using equipment<br>d4154 Maintaining a standing position<br>d4500 Walking short distances
WCL2		d4501 Walking long distances<br>d465 Moving around using equipment<br>b4552 Fatigability<br>b770 Gait pattern functions<br>b750 Motor reflex functions<br>b7602 Coordination of voluntary movements
WCL3		b4552 Fatigability<br>b1300 Energy level<br>b2100 Visual acuity functions,<br>b1440 Short-term memory<br>b6200 Urination<br>b1263 Psychic stability<br>b2801 Pain in body part<br>b5501 Maintenance of body temperature<br>d760 Family relationships

Abbreviations: EDSS, expanded disability status scale; SD, standard deviation; WCL 1, working capacity level 1-MS patients with the lowest working capacity; WCL 2, working capacity level 2, MS patients with intermediate working capacity; WCL3, working capacity level 3-MS patients with the highest work capacity.
